# Supplementary material for: Rapid turnover of life-cycle-related genes in the brown algae
Source: Genome Biol. 2019 Feb 14;20:35. doi: 10.1186/s13059-019-1630-6 (PMC6374913; doi:10.1186/s13059-019-1630-6)
Supplement: Supplementary file 3 — Protein alignments, corresponding Gblocks html file and CDS sequences for the lineage conserved gametophyte-biased genes. (ZIP 2109 kb) [file 13059_2019_1630_MOESM3_ESM.zip › Con_GA_alignments/Ec-06_008070.aa_ali.fasta-gb.htm]

Ec-06\_008070.aa.fasta.trans.aa\_ali.fasta


## Gblocks 0.91b Results

Processed file: **Ec-06\_008070.aa.fasta.trans.aa\_ali.fasta**  
Number of sequences: **2**  
Alignment assumed to be: **Protein**  
New number of positions: **234** (selected positions are underlined in blue)

```
                         10        20        30        40        50        60
                 =========+=========+=========+=========+=========+=========+
Ec-06_008070     MQQEAAAGEARSRTREACWPKQDVKALRQALKDVPAALDKNERFRRVSALVGGSHSKKAC
MSTRG.19000.1    ------------------------------------------------------------
                                                                             


                         70        80        90       100       110       120
                 =========+=========+=========+=========+=========+=========+
Ec-06_008070     YEKYKELKAEAKLKQTTPRTSVSGASPGDSRDRRAANKKKACAGNDGEIISVSPSSAGQQ
MSTRG.19000.1    ------------------------------------------------------------
                                                                             


                        130       140       150       160       170       180
                 =========+=========+=========+=========+=========+=========+
Ec-06_008070     QVEVTSVSTAAGSVTSSTVSGLFSGRSSATTLSSWSSNSADLCARQEQQRQEHIRSMKSN
MSTRG.19000.1    ------------------------------------------------------------
                                                                             


                        190       200       210       220       230       240
                 =========+=========+=========+=========+=========+=========+
Ec-06_008070     ALSSVSSGLPESDMLETQGNFEGDRRGYYPEKSNSRASNDSIDRPTSLAGGGPQTTKHEE
MSTRG.19000.1    ------------------------------------------------------------
                                                                             


                        250       260       270       280       290       300
                 =========+=========+=========+=========+=========+=========+
Ec-06_008070     ELMEVEEVDVEDFCLDEELVVEPRRRVRTTTTTTTPHTRRAEGGRQDWNPAAGNSGAGFH
MSTRG.19000.1    ------------------------------------------------------------
                                                                             


                        310       320       330       340       350       360
                 =========+=========+=========+=========+=========+=========+
Ec-06_008070     RTGQGDSQATTPSSAGGGGGGGSGGLPGRT-AVTEVQANGVRELVFGNPSKRFNDAWREQ
MSTRG.19000.1    -XGAGQ-----PSPARGEVGYGEGKKPGRVSAVTEAEANGVRALVFGDPLKRFNDAWREQ
                            ##################  #############################


                        370       380       390       400       410       420
                 =========+=========+=========+=========+=========+=========+
Ec-06_008070     GFFFCGEDFLRYGLVQAEGGPCGVLAAVQAFVLEDLIFGDCAGCDWRNPKKSQRERSLTS
MSTRG.19000.1    GFYFCAVDGLRYGLVQAEGGPCGVLAVVQAFLLEDMVFGSCAGCEWRNPKRSQRERSLAS
                 ############################################################


                        430       440       450       460       470       480
                 =========+=========+=========+=========+=========+=========+
Ec-06_008070     ALSTIIWSAGDGNTAVLAVSEGGATVQRSSRYRPDGFTERVKLIHVGSRSALEGVVRDNL
MSTRG.19000.1    ALSSIIWRAGDGDTAVLALSEGSATVQRSSRYRPDGFTEKLKLHRAGSRRALAGLVLDNI
                 ############################################################


                        490       500       510       520       530       540
                 =========+=========+=========+=========+=========+=========+
Ec-06_008070     HHYTNRKGQGVILLVYSCVFSRGVDAVRGDMDNSFDEPSPLMASHGYASQELVNLLLVGR
MSTRG.19000.1    SHFREKQGQGVILLVYSCIFARGVEAVRRDMDTGFGEAPTLMASHGYASQGRK-------
                 ##################################################          


                        550       560       570       580       590       600
                 =========+=========+=========+=========+=========+=========+
Ec-06_008070     ARSNVFDGCRVMGGEGGGAKAGAGGKEGCEDGERGQKQGHQKEEDEGGGDDRVVLRGVSE
MSTRG.19000.1    ------------------------------------------------------------
                                                                             


                        610       620       630       640       650       660
                 =========+=========+=========+=========+=========+=========+
Ec-06_008070     RGRVGFLTLFEAYKHVEVGDRLKNPETPIWVVCSESHYSVLFGVDSSIVPPPQPTTASRE
MSTRG.19000.1    ------------------------------------------------------------
                                                                             


                        670       680       690       700       710       720
                 =========+=========+=========+=========+=========+=========+
Ec-06_008070     DSRRQLAGGSGGATAKSAPAAGSSGRGNGGSAASGYGSGGRETDGAEAFDLEYYDGLGRQ
MSTRG.19000.1    ------------------------GWGRSGKAGDDRRAGGR-------------------
                                         #################                   


                        730       740       750       760       770
                 =========+=========+=========+=========+=========+=======
Ec-06_008070     DETIRLTVDQRHDGGDGSPPLTTYNEANDALIPPLDLVIRTRWPEATVDWNGTDPIL
MSTRG.19000.1    ---------------------------------------------------------
```

```
Parameters used
Minimum Number Of Sequences For A Conserved Position: 2
Minimum Number Of Sequences For A Flanking Position: 2
Maximum Number Of Contiguous Nonconserved Positions: 8
Minimum Length Of A Block: 10
Allowed Gap Positions: None
Use Similarity Matrices: Yes
```

```
Flank positions of the 3 selected block(s)
Flanks: [312  329]  [332  530]  [685  701]  

New number of positions in Ec-06_008070.aa.fasta.trans.aa_ali.fasta-gb:  234  (30% of the original 777 positions)
```
